# Up for the tackle? The pelvic floor and rugby. A review

**DOI:** 10.1002/ejsc.12121

**Published:** 2024-05-11

**Authors:** G. M. Donnelly, K. Bø, L. B. Forner, A. Rankin, I. S. Moore

**Affiliations:** ^1^ Cardiff School of Sport and Health Sciences Cardiff Metropolitan University Cardiff UK; ^2^ Private Practice Maguiresbridge, Enniskillen UK; ^3^ Department of Sports Medicine Norwegian School of Sport Sciences Oslo Norway; ^4^ Department of Obstetrics and Gynecology Akershus University Hospital Lørenskog Norway; ^5^ School of Health and Rehabilitation Sciences The University of Queensland Brisbane Queensland Australia; ^6^ Private Practice Brisbane Queensland Australia; ^7^ Sports Medicine Sports Medicine NI Belfast UK

**Keywords:** female athlete, genital hiatus, incontinence, lifespan, perinatal, return to sport

## Abstract

The pelvic floor and its associated disorders are a unique and often overlooked aspect of women's rugby. This review discusses relevant biopsychosocial considerations specific to the pelvic floor and rugby. Pelvic floor disorders can present at any time across the female lifespan but are more prevalent during pregnancy and postpartum. This is due to the substantial physiological and anatomical changes experienced during pregnancy and vaginal childbirth. Consequently, pelvic floor disorders can impact a player's ability to perform, maintain engagement with, or return to, rugby due to symptoms. Players need to be informed, supported, and guided through focused pelvic floor muscle training to condition the muscles and ‘ready’ them for the varied demands of rugby. Health and fitness professionals should understand the risk of pelvic floor disorders across the female lifespan and screen players for symptoms when supporting them to maintain or return to rugby. Rugby players who are symptomatic of pelvic floor disorders should be signposted to specialist services and/or resources to manage their symptoms. Once engaging in rugby training, ongoing evaluation of player load tolerance and implementation of individualized strategies to support managing rugby‐related loads to the pelvic floor should be considered. Finally, surveillance and research focusing specifically on rugby players and pelvic floor function are needed.

## Highlights

- High force‐related activities, such as rugby, challenge the pelvic floor muscles (PFMs) and can increase a player's susceptibility to pelvic floor disorders (e.g., stress urinary incontinence, feeling a bulge inside or outside the vagina)
- Pelvic floor disorders can present at any time across the female lifespan, with pregnancy and childbirth being two commonly reported risk factors for such disorders
- Predisposing (genetics), inciting (pregnancy and vaginal childbirth) and intervening (lifestyle, aging) factors likely influence pelvic floor load tolerance
- PFM training has level 1 evidence to be a first line treatment for urinary incontinence and pelvic organ prolapse in the general female population. We encourage PFM training to be incorporated into rugby training programmes across all ages, levels and roles regardless of whether pelvic floor disorder symptoms are present or not

## 1 INTRODUCTION

The pelvic floor is a unique and often overlooked characteristic of women's rugby. Taking a cupuliform shape, the pelvic floor muscles (PFMs) span the outlet at the base of the pelvis (Bordoni et al., 2022; Herschorn, 2004). Their role is multifactorial and includes (i) maintaining continence (bladder and bowel); (ii) facilitating excretion (bladder and bowel); (iii) supporting the pelvic organs and; and (iv) enabling sexual function (Donnelly et al., 2023). Compromise to any of these roles may lead to signs and symptoms of pelvic floor disorders, also referred to as pelvic floor dysfunction, (PFD) such as incontinence (bladder or bowel), pelvic organ prolapse or pain (Donnelly et al., 2023; NICE, 2021). Symptoms of PFD can occur at any time across the female lifespan and are attributed to combinations of anatomical, physiological, genetic, lifestyle and reproductive factors, rather than a single cause (Delancey et al., 2008). For example, the perinatal period (pregnancy and postpartum) is one factor that increases a woman's predisposition to PFD (Delancey et al., 2008; Donnelly et al., 2023; McCarthy‐Ryan et al., 2024; NICE, 2021). To reduce the risk of provoking, or worsening, symptoms of PFD, women's rugby players must be pelvic floor ‘ready’ for training and performance. It is therefore essential that all supporting professionals, regardless of health or fitness background, are aware of the importance of including focused PFM training within strength and conditioning programing, as well as screening for PFD and signposting to specialist services.

The aim of this review is to discuss the importance of the PFMs across the lifespan of women's rugby players using a biopsychosocial‐informed approach. First, we outline the PFM anatomy, physiology and functioning during rugby. Then, we outline the prevalence and risk of PFD across the female lifespan. We cover key changes to the PFMs during pregnancy, childbirth, postpartum and advancing age along with identifying signs and symptoms of PFD. The final sections will discuss rugby‐specific PFM load tolerance and conditioning, alongside strategies and adjuncts to aid PFD management and optimize successful female engagement in rugby.

## 2 THE FEMALE PELVIC FLOOR COMPLEX

There are many distinct differences in male and female anatomy, one of the main ones is the anatomical structure and role of the pelvic floor. The female PFMs span a wider pelvic outlet than in males and include an additional orifice, the vagina. As a result, the female pelvic floor relies on a more complex system of myofascial connective tissue integrity, neural innervation, vascularization and PFM function to manage the intra‐abdominal pressure (IAP) and external load demands (e.g., ground reaction forces) placed upon it (Donnelly et al., 2023). The PFMs contract during occurrences of elevated IAP (Constantinou et al., 1982) to limit the downward displacement of the pelvic organs (Junginger et al., 2010; Lovegrove Jones et al., 2010; Sapsford et al., 2001; Williams et al., 2022). Typically, PFM activation precedes rises in IAP (Sapsford et al., 2001) and movement‐related load (Okeahialam et al., 2022) in an anticipatory and feed‐forward manner. It is proposed that delayed activation and impaired speed of PFM activity may predispose symptoms of stress urinary incontinence (SUI). However, research is conflicting regarding this mechanism (Kharaji et al., 2019; Leitner et al., 2017; Moser et al., 2018, 2019; Smith et al., 2007), and other factors relating to the anatomical integrity of the pelvic floor complex, discussed below, also influence the continence mechanism.

**FIGURE 1:**
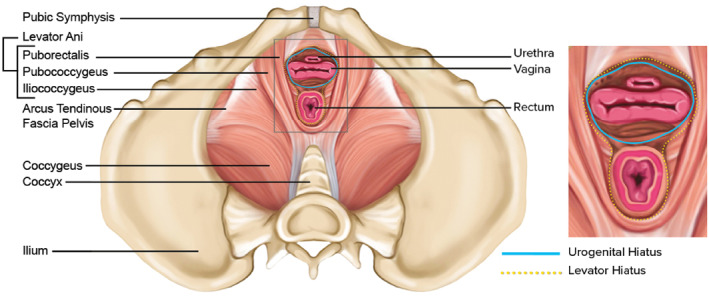
The female pelvic floor muscles and hiatal areas [Adapted from Cheng et al. (2023), Hiatal failure: effects of pregnancy, delivery and pelvic floor disorders on level III factors, International Urogynaecology Journal].

Looking closer at the anatomical design of the PFMs, they can be considered in subsections (Table 1, Figure 1). The levator ani is a group of muscles making up a funnel shaped area in the lower part of the pelvis. From medial to lateral, they include the puborectalis, pubococcygeus and iliococcygeus (Kearney et al., 2004). The pelvic floor is further defined by two spaces, the levator hiatus and urogenital hiatus, both of which are bordered by the pubic symphysis ventrally and the medial borders of the levator ani laterally (Cheng et al., 2023) (Figure 1). The levator hiatus appears as a ‘V’‐shaped area which extends dorsally to the sides of the anorectum and is enclosed by the puborectalis muscle (Cheng et al., 2023; Delancey et al., 1998). The more caudal urogenital hiatus encloses the pubococcygeal portion of the levator ani muscles and extends dorsally from the center of the urethra to the perineal body (Cheng et al., 2023; Delancey et al., 1998).

**TABLE 1:** Overview of pelvic floor anatomy.

Pelvic floor anatomy, risks and consequences	Description
Anatomy and grouping	**Levator Ani muscle group:** Puborectalis, pubococcygeus and iliococcygeus
	**Urogenital diaphragm:** Deep transverse perineal muscles, constrictor of the urethra and internal and external fascial coverings
	**Urogenital hiatus:** Bounded laterally by the medial margins of the levator ani, specifically the pubococcygeal portion, it extends from the center of the urethral meatus to the posterior midline of the hymen
	**Levator hiatus:** ‘V’‐shaped medial component of the levator ani muscle, corresponds with the puborectalis muscle
Role	Support and maintain the position of the pelvic organs
	Support and maintain the continence mechanism for the bladder and bowel.
	Normal excretion of urine and feces
	Sexual function
Potential pelvic floor risk to the postpartum player	Sustained stretch and loading of the muscles during pregnancy and childbirth, alongside associated tissue trauma
	Hiatal ballooning leading to pelvic organ prolapse and descent of the perineal structures
	Potential compromise to function
	Birth induced injury to the pubococcygeal muscle portion of the levator ani muscles is strongly associated with pelvic organ prolapse and descent of the perineal structures
	Potential for pelvic floor muscle avulsion injury
	Birth induced pudendal nerve injury
Potential consequence of pelvic floor risk to return to rugby	Slow or delay rehabilitation progression
	Delay return to training or matches
	Limit time played during training/matches
	Limit sport performance
	Negatively impact player quality of life and sport enjoyment
	Negatively impact player mental health

While focus has been on the deep layer of PFMs, anatomical understanding should extend to the role of connective tissue in pelvic organ support and the continence mechanism, particularly the endopelvic fascia. This layer of dense, fibrous connective tissue attaches the bladder, uterus, vagina and rectum to the pelvic walls via fascial attachments with the arcus tendinous fascia pelvis and the medial portion of the levator ani (Roch et al., 2021). One of the endopelvic fascia's recognized roles is to provide tensile strength to the anterior pelvic structures (e.g., urethra, bladder) (Ashton‐miller et al., 2007). In addition to compromised pelvic organ support via changes to the endopelvic fascia, poor levator hiatal closure may also negatively impact the continence mechanism as the habitual stiffness of the PFMs will be reduced allowing greater downward movement of the pelvic viscera and possible disruption of the urethral closure mechanism. Specifically, if the PFMs are situated adequately and the levator hiatus remains closed, the stiffness in the tissues limits excessive downward movement and ballooning of the hiatus (Bø, 2020).

## 3 THE PELVIC FLOOR DURING RUGBY

The mechanisms and behavior of the PFMs prior to, or during, movement are not well understood (Bø et al., 2020). One research group used a novel wireless intravaginal pressure transducer during a range of movements that required regulation of effort (Shaw et al., 2014) and analyzed the IAP of the activities relative to each individual's maximum potential IAP, determined via seated straining (Valsalva) (Dietze‐Hermosa et al., 2020). Walking generated mean IAPs of 21% of maximum IAP, whilst running, generated 56% of maximum IAP, with some individuals reaching as high as 203% (Dietze‐Hermosa et al., 2020). However, findings from studies to date regarding IAP or PFM activity should be considered with caution, as intravaginal measurement devices are likely to move and pick up artifact from surrounding muscles and tissues. Further, the dynamic forces the PFMs must be able to tolerate during movements are not well understood and are likely to be highly individualized. Valid measuring systems to quantify IAP and PFM activity during vigorous physical activities, such as those involved in rugby, need to be developed and investigated.

Several factors are likely to influence the demands and loads placed upon the PFMs, such as the ability of the body to attenuate force. For instance, a player's running technique will affect the ground reaction forces produced (Breine et al., 2017) and it is not known how much the lower limb attenuates ground reaction forces prior to reaching the PFMs. In addition to ground reaction forces, IAPs exert a load onto the PFMs. What constitutes a high magnitude of IAP appears to depend upon an individual's capacity to generate and manage IAP.

In the context of rugby, many of the movements a player is exposed to result in large forces (Nagahara et al., 2021; Trewartha et al., 2015; Usman et al., 2011) being transferred either directly to the abdomino‐lumbopelvic region (e.g., being tackled with contact to this region) or indirectly from load transferred through the body (e.g., force transferred through the shoulders during a scrum). These forces are likely to produce high IAPs (Kawabata et al., 2010; Nagahara et al., 2021) and subsequently direct high forces toward the PFMs (Bø et al., 2020; Shaw et al., 2014). To withstand this pressure, it is thought that PFM activity must increase from baseline to anticipate and accommodate the demands of movement or force (Moser et al., 2018). For example, the PFMs will theoretically be required to anticipate a tackle and accommodate forces received whilst being tackled. The ability to manage and tolerate these loads is likely to be player‐specific and influenced by several factors discussed further in this paper. Furthermore, symptomatic players may be able to tolerate the load in some movements but not others, and the ability to tolerate load will most likely reduce with training and match‐related fatigue (Thomaz et al., 2018).

## 4 PELVIC FLOOR DYSFUNCTION

### 4.1 Symptoms and prevalence

Impairment to the function of the PFMs may result from compromised connective tissue support, compromised innervation, PFM weakness or injury. Symptoms of PFD (Table 2) relate to the impairment of any role that the PFMs are involved in. Players with PFD may present with specific symptoms that relate to a subset of the anatomical structures discussed previously. Each structure should therefore be considered in its role in pelvic organ support and continence and contextualized by individual whole‐system considerations, such as aging and lifestyle factors (Delancey et al., 2008).

**TABLE 2:** Considerations for symptoms, cues, training dosage, progression, adjuncts and signposting for pelvic floor disorders.

Consideration	Recommendation
Symptoms of PFD	Urgency, frequency and/or incontinence (bladder and/or bowel, including flatus)
	Heaviness, pressure, bulge, dragging in the vaginal area
	Issues emptying bladder or bowel (e.g. obstructive defecation, post‐void residual)
	Recurrent urinary tract infections
	Pelvic floor pain, dyspareunia, sexual dysfunction
	Vaginal dryness while lactating or associated with age‐related changes
Cues for pelvic floor muscle training	“Stop gas escaping”
	“Squeeze and lift around the urethra, vagina and rectum”
	“Stop the flow or urine mid‐flow”
	“Close a zip back passage to front passage”
	“Close the vagina”
	“Close the anus”
Training dosage and progressions	**Step 1:** Basic focused pelvic floor training program of 8–12 MVC's aiming to hold up to 10 s each. Repeat 2–3 times per day if symptomatic
	**Step 2:** The pelvic floor can also be recruited in preparation for a leakage‐provoking event (the “knack”)
	**Step 3:** Vary positions of pelvic floor training to reflect upright and task specific activities in rugby
	**Step 4:** Graded exposure to resistance/weights training beginning with static, closed chain options and progressing to dynamic options. Monitor for symptoms of PFD as weight and difficulty of training increase. Modify and regress as indicated
	**Step 5:** Graded exposure to impact activities, for example, running and jumping. Monitor for symptoms of PFD. Modify and regress as indicated
	**Step 6:** Graded exposure to spontaneous load through tackle training. Monitor for symptoms of PFD. Modify and regress as indicated
	Step 7: Return to simulated match play. Monitor for symptoms of PFD. Modify and regress as indicated.
	Step 8: Return‐to‐sport. Monitor for symptoms of PFD as training and match volume increase.
	**Step 9:** Long term adherence—include focused pelvic floor muscle training 1–2 times per week. May be part of a wider training program.
Adjuncts to pelvic floor muscle training and symptom management	Vaginal pessaries for stress urinary incontinence and/or pelvic organ prolapse
	Targeted pelvic floor compression garments
	Femtech
Indication for signposting and onward referral	No improvement in symptoms despite adhering to regular pelvic floor muscle training
	Unsure how to locate and train the pelvic floor despite information and cues
	Symptom progression
	Persisting pelvic pain
	Suspected medical issues (e.g. urinary tract infection, vaginal infection, postpartum complication, poor healing postpartum, presence of red flags)

Abbreviations: Femtech, female technology; MVC's, maximum voluntary contractions; PFD, pelvic floor disorders/pelvic floor dysfunction.

Within the general population, it is estimated that one in three women experience urinary incontinence, up to one in 10 experience fecal incontinence and up to one in every two women have some degree of pelvic organ descent (NICE, 2021; Woodley et al., 2020; Brown et al., 2019). High force‐related activities, such as rugby, challenge the PFMs and can increase a player's susceptibility to PFD symptoms (Almeida et al., 2016; Campbell et al., 2023; de Mattos Lourenco et al., 2018; Delancey et al., 2008; Donnelly et al., 2023; McCarthy‐Ryan et al., 2024; Moore et al., 2021; Sandwith et al., 2021). In a cohort of 95 female University varsity rugby players, 54% of players leaked urine of which 90% leaked when competing and 88% leaked when being tackled or hit (Sandwith et al., 2021). A larger study (*n* = 396) of women's Rugby Union community to national level players across four Nations found 63% had general SUI and 43% leaked during rugby. The most prevalent symptom‐inciting events were the tackle, running and jumping. Identified risk factors for SUI during rugby included being postpartum, having a higher body mass index, being a forward and having a lower level of playing experience (McCarthy‐Ryan et al., 2024). Furthermore, reporting constipation was associated with rugby‐related SUI (McCarthy‐Ryan et al., 2024), which may result from persistent constipation increasing the strain on the PFMs over time (Delancey et al., 2008). This highlights the interaction of co‐existing symptoms and behaviors and the need to consider the wider whole‐system factors in individual player presentations (Donnelly et al., 2022).

To date, studies have determined the prevalence of PFD in women's rugby players as a collective, but we do not understand its prevalence or impact in subpopulations such as perinatal players. This is due to the lack of surveillance and the overall paucity of research investigating female‐specific health domains (Moore et al., 2023) with previously only McCarthy‐Ryan and colleagues (McCarthy‐Ryan et al., 2024) including postpartum rugby players. Consequently, there is a need for research to investigate how the risk factor of being postpartum influences the prevalence and severity of PFD in women's rugby (Heyward et al., 2022; McCarthy‐Ryan et al., 2024).

### 4.2 Predisposing, inciting and intervening factors

**FIGURE 2:**
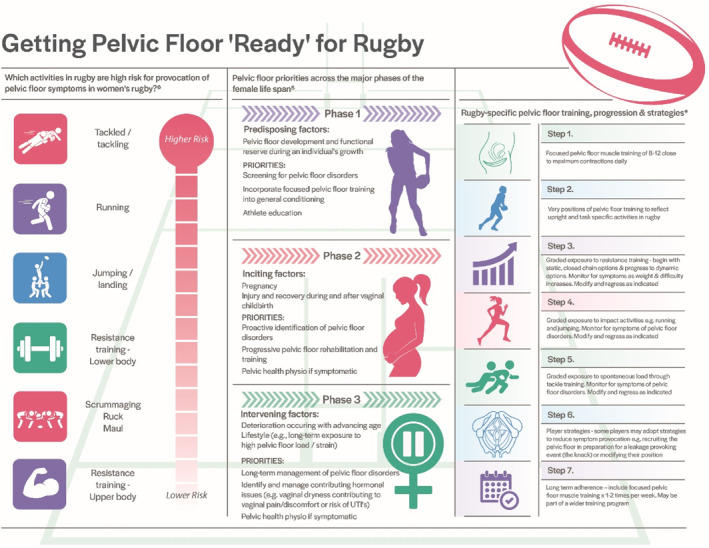
Getting Pelvic Floor ‘Ready’ for Rugby. *These recommendations are based on exercise prescription principles and research on the general population. Research specific to women's rugby players across all ages and levels is needed to better inform this population.

De Lancey and colleagues (Delancey et al., 2008; DeLancey et al., 2024) outline three major phases across the female lifespan where PFM function and dysfunction relate (Figure 2). *Phase 1* is *predisposing factors (development and functional reserve during an individual's growth)*; *Phase 2* is *inciting factors (variations in the amount of injury and potential recovery that occur during and after vaginal childbirth)*; and *Phase 3* is *intervening factors (lifestyle, deterioration occurring with advancing age*). Each of these will be discussed briefly below.

#### 4.2.1 Phase 1—Predisposing factors

##### Development and functional reserve

It is proposed that the development of the PFMs early in the female lifespan will influence an individual's functional reserve and thus future likelihood of PFD (Delancey et al., 2008). Development and functional reserve of the PFMs are likely to be determined by several factors, including genetics, lifestyle, diet and environment (Delancey et al., 2008). For example, high body mass index and constipation are established risk factors for PFD (NICE, 2021). Early education about physical activity (Chief Medical Officer, 2019), lifestyle, the anatomy and function of the pelvic floor and how to locate and train the PFMs during adolescence is advocated to promote improved development and functional reserve (DeLancey et al., 2024; NICE, 2021). However, this theory needs to be investigated further, especially in the context of sports‐specific PFM function.

#### 4.2.2 Phase 2—Inciting factors

##### Pregnancy and childbirth

The PFMs are exposed to an increasing magnitude of load from the growing uterus as a pregnancy progresses. Accordingly, the dimensions of the PFMs, and associated hiatal areas (Figure 1), increase in size to prepare for childbirth, regardless of the mode of delivery (Stær‐Jensen et al., 2013). The levator hiatal area enlarges from the first to the third trimester of pregnancy at rest, on PFM contraction, and during bearing down, by an average of 13%, 10% and 29%, respectively (Cheng et al., 2023). These normal, anticipated changes are important to ready the body and facilitate potential vaginal childbirth. However, they also carry a risk for negative implications associated with increasing hiatal distensibility, especially when subsequent vaginal childbirth is considered too. For women who experience vaginal childbirth (approximately 80% of worldwide childbirths) (Betran et al., 2021), major changes to the PFMs occur including compression and stretching of PFM soft tissues that extend beyond the capacity of most other muscles in the body. Specifically, the baby's head stretches the levator ani muscle, generating predominantly passive forces in the tissue of the levator hiatus as it passes through the PFMs (Tracy et al., 2016). For non‐pregnant muscle tissue, the maximum non‐injurious stretch (lengthening) that a muscle can undergo before injury is estimated to be 150% of its original length (Brooks et al., 1995). Comparatively, the PFMs experience a stretch much higher than this during vaginal childbirth, up to 250% their original length (Krofta et al., 2017). Therefore, birth‐induced injury to the levator ani muscles during vaginal childbirth includes over‐stretching (microtrauma) or tearing and avulsion (macrotrauma) and varying degrees of either are considered to occur in all vaginal deliveries. The prevalence of PFM injury (macrotrauma) specifically from vaginal childbirth ranges from 18% to 41% (Cardozo et al., 2023; Miller et al., 2015). Additionally, obstetric anal sphincter injuries (also known as third and fourth degree perineal tears) have been reported in up to 8% of vaginal deliveries in the UK (Thiagamoorthy et al., 2014). A recent meta‐analysis of risk factors for perineal laceration covering all degrees reported risks to include primiparity, instrumental delivery (particularly forceps) and newborn birthweight (Pergialiotis et al., 2020).

Birth‐induced injuries are associated with descent of the pelvic organs and perineal structures (Cheng et al., 2023; Clark et al., 2010; DeLancey et al., 2012). Specifically, the distensibility of the levator ani causes an increase in the cross‐sectional area of the levator hiatus. Excessive distensibility, termed hiatal ballooning, is associated with the occurrence of pelvic organ prolapse (Siahkal et al., 2021; Xuan et al., 2019). In fact, this region is considered to have the largest potential hernia portal in the human body (Xuan et al., 2019). Most of the recovery in size of the levator hiatus occurs within 4–6 months after delivery (Cheng et al., 2023). However, it does not return to pre‐pregnancy size, and this recovery time is further complicated depending on the degree of trauma at the time of delivery (Bø et al., 2022; Cheng et al., 2023; Stær‐Jensen et al., 2015). Whilst the transition into and beyond pregnancy is a normal bodily process, it can impair the function of the PFMs and lead to reduced sport participation, time loss from sport and career cessation (Dakic et al., 2021; McCarthy‐Ryan et al., 2024). Unlike return‐to‐sport following injury, which is widely discussed in the sports medicine and science literature, return‐to‐sport after pregnancy and childbirth have been largely overlooked. The physiological changes to the PFMs during pregnancy warrant consideration and purposeful strengthening and conditioning, even in players who experience childbirth via caesarean section. Given the increased size of the levator hiatal area by the third trimester of pregnancy (Bø et al., 2015), reconditioning to encourage a return toward baseline size, resting tone and strength is paramount, especially in the context of the load tolerance required of the PFMs during rugby. In most countries, a large focus is placed on PFM training during pregnancy, as early structured PFM training can prevent the onset of urinary incontinence in mid and late pregnancy (Woodley et al., 2020) and limit the perceived symptoms of pelvic organ descent (Hagen et al., 2014, 2017). Evidence has shown that women with enhanced understanding of the PFMs are 57% less likely to develop urinary incontinence (Cardoso et al., 2018). Consequently, from a PFD prevention perspective, rugby players across all ages and levels should be educated on the anatomy and function of the PFMs.

#### 4.2.3 Phase 3—Intervening factors

##### Lifestyle

The type of sport women longitudinally engage in may expose the PFMs to different loads that appear to effect PFM morphology (Menezes et al., 2023) and contribute to the risk of PFDs. That is, high force activities such as rugby may alter morphology in a way that increases the risk. When compared to non‐active/low impact exercising controls, competitive athletes engaging in high‐impact training for over 5 years exhibit greater levator hiatal width (Kruger et al., 2007; Menezes et al., 2023) and distensibility as well as higher degrees of pelvic organ descent (Kruger et al., 2007). Further, engaging with 30 min of exercise 3 times per week during pregnancy leads to a larger hiatal area at rest and during PFM contraction compared to not exercising in the third but not the second trimester (Bø et al., 2015). These studies highlight the potential changes that can present in nulliparous athletes through exposure to sports like rugby, with pregnancy possibly modifying this interaction. Exposure to high‐impact sports can also lead to acute changes in hiatal dimensions, but this is consistent in runners with and without SUI (Bérubé et al., 2023) and the long‐term consequences are not fully understood.

##### Deterioration occurring with advancing age

With player longevity being an important consideration as more women potentially take up rugby in later life or continue participating for longer (e.g., beyond motherhood), an understanding of the age‐related deterioration in PFMs is needed. Normal decline of the PFMs is expected with age‐related changes including increased fiber length (Alperin et al., 2016; Cook et al., 2017), fibrosis (Alperin et al., 2016) and a reduction in muscle mass and connective tissue tensile strength associated with the decline in estrogen (Chidi‐Ogbolu et al., 2018). The decline of the PFMs across the lifespan may also be influenced by several factors including obesity, arduous occupations or chronic constipation (DeLancey et al., 2023; Jackson et al., 2022; NICE, 2021). For women, menopause may interact with the risk of PFD due to associated hormonal changes during this age‐related transition (Angelou et al., 2020; Peinado‐Molina et al., 2023). However, to the authors' knowledge, no studies have examined the influence of hormonal changes or the transition into menopause in women's rugby players. Raising awareness of the impact that advancing age and the transition into menopause can have on an individual's PFMs and associated function is important.

## 5 RECONDITIONING THE PELVIC FLOOR

Like other muscle groups, the PFMs can be trained via targeted strengthening and conditioning and there is consistent evidence that PFM training can induce muscle hypertrophy, reduce the levator hiatal area and improve the symptoms associated with PFD (Cacciari et al., 2021, 2022; Hagovska et al., 2022; Hoff Brækken et al., 2010). Regardless of the predisposing, inciting and or intervening factor(s), symptomatic players should engage in appropriate PFM training. For example, postpartum rugby players should be informed about commencing PFM training as soon as possible following childbirth. Following vaginal delivery, even in the presence of perineal tears and stitches, PFM training can gently commence. Medically complicated deliveries or any delivery that results in a catheter in situ will delay PFM training until the catheter is removed. Based upon exercise prescription principles, rugby players should follow general strength training recommendations (Garber et al., 2011). In terms of the PFMs, strength training dosage should aim for fatigue of the PFMs by the end of the set(s). This could involve engaging in focused PFM training of 3 sets of 8 to 12 close to maximum PFM contractions repeated daily (Bø et al., 2024; Fleck, 2004) during early rehabilitation and reducing to one to two times per week for maintenance as recovery progresses or symptoms resolve (Bø et al., 2020; Garber et al., 2011). However, more research is required in relation to PFM training dosage in athletic populations such as rugby and players will have differing reconditioning needs dependent upon individual predisposing, inciting and intervening factors. Where possible, PFM reconditioning should be informed and guided by a pelvic health physiotherapist (sometimes referred to as women's health physiotherapist) (Pelvic Obstetric and Gynaecological Physiotherapy, 2022). Table 2 offers useful prompts to help rugby players understand how to locate and train their PFMs as well as guidance on load progression, adjuncts and when to signpost or refer onwards for specialist support.

### 5.1 Vaginal tissue health

Lower levels of estrogen in the female body can increase vaginal dryness and sensitivity (Goncharenko et al., 2019). These vaginal changes may have a negative impact upon sexual function, player comfort and ability to train or play matches. There may also be an increased risk of health problems (e.g., urinary tract infections) (Goncharenko et al., 2019). Lower estrogen levels are associated with lactation (Calik‐Ksepka et al., 2022) and aging (menopause) (Angelou et al., 2020) and therefore breastfeeding and perimenopausal rugby players are likely to be impacted. Player support staff should be aware of these challenges and signpost players to appropriate healthcare professionals for management (e.g., General Practitioner, Gynecologist) where vaginal moisturisers or localized estrogen may be indicated.

### 5.2 Psychological wellbeing

Focusing beyond localized muscle dysfunction or tissue trauma, wider whole‐system factors associated with mental health, psychosexual trauma (Karsten et al., 2020) or birth trauma (Greenfield et al., 2016) can negatively impact PFM function. The reverse is also true whereby psychological wellbeing (across the female lifespan) is negatively impacted by PFD (NICE, 2021). The transition into motherhood, for example, can be challenging for many women, with up to 40% experiencing perinatal mental health problems such as postpartum depression (Wang et al., 2021). In fact, postpartum depression is three times as prevalent in women with PFD than women without it (Mazi et al., 2019). Furthermore, their experience of interactions and events directly related to childbirth may cause overwhelming distressing emotions and reactions, leading to short and long‐term negative implications on their health and wellbeing (Leinweber et al., 2022). This means that both physical and/or psychologically traumatic experiences during childbirth (“birth trauma”) can incur ongoing psychological consequences including catastrophizing and compromised mental health (Leinweber et al., 2022; Shorey et al., 2022). Physical symptoms of birth trauma include birth‐related tissue injury, reduction in functional capacity, fatigue and persistent postpartum pain (Daly et al., 2017; Kainu et al., 2010; Taghizadeh et al., 2013). Consequently, postpartum players experiencing birth trauma may be disengaged from adhering to postpartum rehabilitation advice, such as PFM training. They may also perceive the area to be vulnerable or fragile, impacting their ability to participate in reconditioning and progressive loading and potentially increasing the risk for fear of movement. Awareness of these conditions along with regular screening and monitoring should be in place so that players can be signposted to specialist psychological support as necessary. A validated tool, such as the Edinburgh Postnatal Depression Scale (Gollan et al., 2021) or the Clinician Administered Post‐Traumatic Stress Disorder Scale (de Graaff et al., 2018), can be used to screen players as appropriate.

## 6 PELVIC FLOOR LOAD TOLERANCE

An integral part of rugby‐specific training includes strength training. Progressive and functional strength training helps prepare players for the demands of rugby, which involves player‐to‐player contact and multidirectional movements (Dane et al., 2022). The tackle is the most frequent contact event in Rugby Union and Rugby League, with a mean of 280 (West et al., 2022) and 512 (Cummins et al., 2020) tackles per game, respectively. In both Union and League matches, player‐to‐player contacts are higher in forwards than in backs (Cummins et al., 2020; Woodhouse et al., 2021). Additionally, players perform high‐speed running, accelerations and decelerations, change of directions, lineout lifts with high‐impact landings, as well as skill‐based events such as catching, passing and kicking the ball. Players must successfully execute these technical actions numerous times over the course of a game, whilst under pressure and experiencing fatigue (Dane et al., 2022). The frequency of these contact and non‐contact skills is dependent on playing position, meaning strength requirements are position‐specific.

Training requirements for women's rugby should include focused PFM training (Table 2, Figure 2) (Donnelly et al., 2023) alongside whole‐body resistance training. Whole‐body resistance training is an important part of conditioning a player for the demands of rugby. In the context of the PFMs, whole‐body resistance training may enable insight into PFM load tolerance. If symptoms of PFD are only provoked as resistance training is progressed, it highlights the need for further focused PFM training and/or review by a pelvic health physiotherapist. Whilst there is limited research examining the effect of whole‐body resistance training and the response of the PFMs, lifting higher loads is considered to increase the IAP placed upon the PFMs (Bø et al., 2020), which may increase the risk of PFD. This may explain the higher prevalence of PFD symptoms, (specifically urinary and anal incontinence) observed in Norwegian female powerlifters (Skaug et al., 2022) compared to the general population (NICE, 2021). In rugby, nulliparous and parous women report SUI to be prevalent (42%) during lower body strength training (McCarthy‐Ryan et al., 2024). However, when considering the loads associated with strength training, women lifting heavy weights (>50 kg) do not report more symptoms of pelvic organ prolapse compared to women lifting lower weights (<15 kg) (Forner et al., 2020). Additionally, acute exposure to heavy lifting does not appear to have negative effects on PFM strength (Skaug et al., 2023). Further research is needed to understand the prevalence of PFD in rugby players exposed to acute and chronic heavy resistance training as well as the long‐term implication of whole‐body resistance training on the PFMs.

The Valsalva strategy that is often necessary to lift heavy loads and prepare for rugby demands has been shown to produce high levels of IAP (Cummins et al., 2020; Dane et al., 2022; Eliasson et al., 2008; West et al., 2022; Woodhouse et al., 2021). Strenuous activity and sport can result in SUI even in nulliparous athletes (Eliasson et al., 2008; Kruger et al., 2007), with the risk for SUI and compromised pelvic organ support further increased in vaginally parous athletes. Studies comparing the impact of pregnancy and vaginal childbirth on the PFMs found that women who delivered vaginally had a greater hiatal area on Valsalva compared to nulliparous women (Cattani et al., 2022). This lack of support and low stiffness in the PFM tissues can compromise the continence mechanism and pelvic organ support. This is supported by Howard and colleagues (Howard et al., 2000) who found that SUI parous women demonstrate greater bladder neck descent during a cough than continent parous women. A non‐significant lower stiffness was also observed for the SUI group. Teams supporting postpartum players to return to rugby should be aware of the increased risk of PFD associated with vaginal delivery and manage players accordingly.

It is not only the mode of delivery but also the likely generalized deconditioning incurred during the perinatal period that should be considered for rugby players who need to cease contact‐related rugby activities during pregnancy and have individualized levels of associated rest from rugby postpartum (World Rugby, 2024). Such generalized deconditioning may also present following severe injuries, such as anterior cruciate ligament rupture. As a result, most postpartum rugby players will have reconditioning needs to prepare for the physical demands, skills and high‐volume of contacts required during rugby (Dane et al., 2022). Graded exposure to postpartum strength training is encouraged and should center around symptom‐free training (Donnelly et al., 2024; World Rugby, 2024). Evidence regarding appropriate timeframes to return‐to‐sport postpartum is lacking. Based on clinical and exercise professional expert opinion, a Delphi study recommended a minimum timeframe of 3–6 weeks relative rest prior to returning to running postpartum (Christopher et al., 2023) and recent World Rugby guidelines indicate 16 weeks postpartum as a minimum time for returning to rugby matches (World Rugby, 2024). Individual timeframes will vary based on whole‐system factors (e.g., delivery mode, psychological readiness to return) and more detail is provided by Donnelly and colleagues (Donnelly et al., 2024) in this special issue.

When undertaking rugby training across the female lifespan, symptoms of PFD should be appropriately identified, managed and addressed according to individual needs. If symptoms are present, strength training should be modified to reduce the training load by way of lowering the weight, reducing the number of repetitions or modifying the resistance exercise or position to reduce the PFM load. If PFM weakness has been assessed as a contributing factor to PFD symptoms, focused PFM training may need to be adjusted to address this. Strategies which may assist PFM function during return to load exposure are discussed later.

Traditionally, sports medicine and science research have focused on the training load accumulated by a player (acute and chronic volume) when assessing injury risk (Blanch et al., 2016) and return‐to‐sport load (Ritchie et al., 2017). However, for a postpartum player as one example, load considerations must go beyond training volume and perceived effort. Specifically, the new life demands brought on by the role of motherhood may mean that atypical daily activity loads accumulate (e.g., lifting, carrying a baby, pushing the pram up hills). Additionally, the wider biopsychosocial challenges that may present after having a baby (e.g., sleep deprivation, lactation, inadequate nutrition, mental health) will ultimately influence a player's recovery, conditioning and tolerance for training. Therefore, psychosocial loads should also be considered when evaluating PFM load tolerance. Collectively, non‐rugby, daily physical activity loads, biopsychosocial loads and additional rugby loads will contribute to a player's readiness and return to rugby training and game play.

## 7 PELVIC FLOOR STRATEGIES FOR RUGBY PLAYERS

How different players tolerate the same PFM load may be explained by predisposing, inciting and intervening factors (Delancey et al., 2008; DeLancey et al., 2023) discussed earlier in this review. For example, variation in player tolerance may also be relative to the functional reserve each player achieved during growth and development (Delancey et al., 2008). Players who progress to superior PFM strength and tolerance to load during earlier life are likely to have more functional reserve and resilience to inciting (pregnancy and childbirth) and intervening (lifestyle and age‐related decline) factors which increase the risk of PFD. Further, player strategies employed during rugby may add to the combination of factors influencing a player's risk including behavioral and non‐behavioral strategies.

### 7.1 Behavioral strategies

Symptoms of PFD suggest that a player is not tolerating the load being placed upon the PFMs, yet many players continue to play when experiencing symptoms (McCarthy‐Ryan et al., 2024; Sandwith et al., 2021). Where a player is symptomatic, symptoms may be reduced or overcome by implementing rugby‐specific strategies. Specifically, rugby players who leak urine during contact‐related activities report modifying their body position (technique) and reducing the number of contact activities they engage in (McCarthy‐Ryan et al., 2024). Whereas the most common strategy for non‐contact activities was reducing movement speed or height jumped as well as modifying technique and reducing the number of non‐contact activities engaged.

Some players may use Valsalva during strenuous rugby movements (e.g., a scrum) to achieve the required magnitude of strength, as Valsalva results in increased trunk stiffness (Hughes et al., 1989) and increases IAP (Blazek et al., 2019). This associated increase in IAP (internal load) can elevate the load placed onto the PFMs and therefore may increase the risk of PFD symptoms due to the repetitive exposure to larger IAPs and potentially higher loads being transferred to the PFMs. However, further research is required to understand the consequences of the Valsalva in female athletes, including rugby players. Furthermore, recently published expert opinion suggests that strategies, including (i) optimizing the technique of abdominal bracing to optimize abdominal cavity force distribution and (ii) engaging in subthreshold training while pelvic floor capacity is increased, may be worthwhile approaches for players who achieve performance benefits with the Valsalva (Prevett et al., 2024).

Another strategy from clinical practice is changing from a Valsalva strategy to purposively breathing, grunting or vocalizing during effort. This has been shown to reduce IAP in weightlifters compared to that generated during Valsalva strategies (Hagins et al., 2006) and therefore may theoretically reduce the IAP forces directed toward the PFMs. Providing players with possible strategies allows them to choose the most effective strategy for reducing *their* symptoms, as well as potentially helping to reduce the cumulative impact of loads on the PFMs; however, research is needed to substantiate this. Irrespective of these rugby specific strategies, PFM training should be included as first line management where load tolerance deficits are identified. Additionally, players should be signposted to a pelvic health physiotherapist for individualized evaluation of their rehabilitation needs.

### 7.2 Non‐rugby behavioral strategies

Players may try to manage symptoms of PFD by altering their bladder emptying behavior and fluid intake, which can lead to the development of further symptoms of PFD. For example, a rugby player who suffers from SUI may try to reduce symptoms by restricting their fluid intake (Culleton‐Quinn et al., 2022; Johnston et al., 2023) and frequently emptying their bladder before and during training (Culleton‐Quinn et al., 2022). This reduction in fluid intake and simultaneous purposeful increase in frequency of voiding is hypothesized to influence the risk of developing further urinary symptoms. For example, urinary frequency, urgency and urge incontinence could develop due to conditioning of the bladder to smaller, more concentrated urine volumes and disruption to the normal urge capacity prior to voiding. However, such behavioral causes of overactive bladder presentations have yet to be supported by research. Nonetheless, it is important that evidence‐based guidance is provided to rugby players regarding maladaptive bladder behaviors and best practice strategies and behaviors (e.g., adequate fluid intake, PFM training) (Booth et al., 2023).

## 8 ADJUNCTS AND ADDITIONAL CONSIDERATIONS

Available options to help minimize the disruption experienced and adjustment required as a consequence of PFD should be communicated to players across all ages and levels. This extends to wider roles involved in rugby (e.g., female coaches and match officials). One example includes digital applications within female technology, which can prompt reminders, direct technique and reinforce educational guidance on PFM exercises. However, the evidence informed quality and efficacy of some digital applications are low (Jaffar et al., 2022; Sudol et al., 2019), and therefore, recommendations of specific applications should be carefully informed.

Players experiencing symptoms of SUI or pelvic organ prolapse may benefit from trying an intravaginal support or continence device (such as a vaginal pessary) to facilitate return to training and match‐play and sustain symptom management. Pessaries for SUI and pelvic organ prolapse have the potential to fully manage and alleviate symptoms (Donnelly et al., 2023; NICE, 2021), thereby allowing women's rugby players to continue engaging with training and return‐to‐sport. Players may also benefit from wearing targeted compression garments during training and matches. Compression garments targeting the PFMs are relatively new and have received limited research attention. Studies have highlighted that they reduce SUI and positively influence the perception of PFD symptoms (Ninomiya et al., 2014; Okayama et al., 2019), which may subsequently improve a symptomatic player's confidence to exercise. Additionally, consideration should be given to the practicality, color, and comfort of player uniform as a study involving commonwealth athletes (that included a small sample of women's rugby players) identified sports involving fitted uniforms (e.g., gymnastics) created worry about an incontinence pad being visible (Johnston et al., 2023). Therefore athletes, including rugby players, need to be able to wear comfortable clothing that facilitates discrete wearing of incontinence or menstrual products.

## 9 PELVIC FLOOR SURVEILLANCE AND FUTURE RESEARCH

Appropriate injury and illness surveillance should be in place to capture any PFD. Pelvic floor health and postpartum are two female athlete health domains recently proposed as key considerations when undertaking female injury and illness surveillance (Moore et al., 2023). Moore and colleagues (Moore et al., 2024) in this special issue highlight that in a rugby context, a non‐time‐loss health problem definition should be implemented to enable PFD data to be recorded as rugby players suffering from PFD do not necessarily stop training or discuss symptoms with their coaches, but rather modify training activities and continue playing (McCarthy‐Ryan et al., 2024). Annual baseline screening as well as ongoing screening during postpartum rehabilitation is advocated for PFD due to the accumulated and changing rugby exposure experienced by players. Whilst validated questionnaires for PFD can be used and allow comparisons between different populations, such questionnaires are general and not sport‐specific. The first sports‐specific screening tool for PFD has been developed (The PFD‐SENTINEL) (Giagio et al., 2023) and may assist with player screening and surveillance; however, it has not yet been validated.

Drawing on postpartum return to running evidence (Christopher et al., 2023; Donnelly et al., 2020; Moore et al., 2021), for the postpartum rugby player, it may be appropriate to ask about urinary and fecal incontinence, flatus incontinence, feeling of vaginal heaviness or, a bulge inside or outside the vagina and musculoskeletal pelvic pain during rugby‐specific activities (e.g., being tackled, tackling, running, rucks and scrums) to determine the intervening event and inform management strategies. Research specific to the PFMs and rugby, which focuses on better understanding the normal behavior of the PFMs during exercise as well as prevention, childbirth related injury and physical performance, is needed to better understand and guide PFM preparation and recovery in anticipation for playing rugby.

## 10 SUMMARY

Awareness and recognition of the changes the PFMs go through during development, pregnancy, childbirth and advancing age is the first step to understanding how to support players to be PFM ‘ready’ for engaging in rugby. The next step is understanding signs and symptoms of PFD and being able to evaluate player load tolerance, considering physical and psychosocial (external and internal) loads. We have outlined several strategies that rugby players may use to reduce or overcome symptoms of PFD, whilst recommending that PFM training is undertaken by all rugby players as a prophylactic approach and as a first line treatment for PFD. Being PFM ready for rugby will require symptomatic players to engage in a reconditioning program with progressive loading. By applying the steps and recommendations identified in this review, enhanced rehabilitation support can be provided to all women's players by health and fitness professionals, which can minimize the risk and or symptoms of PFD and enable continued rugby participation across all age, roles and levels of the game.

## CONFLICT OF INTEREST STATEMENT

ISM receives funding from the Welsh Rugby Union and World Rugby and is an advisor to the Rugby Player's Association Women's Welfare group.

## COMPETING INTEREST STATEMENT

The authors report there are no competing interests to declare.
